# Polyamine Ligand-Mediated Self-Assembly of Gold and Silver Nanoparticles into Chainlike Structures in Aqueous Solution: Towards New Nanostructured Chemosensors

**DOI:** 10.1002/open.201300023

**Published:** 2013-08-02

**Authors:** Adrián Fernández-Lodeiro, Javier Fernández-Lodeiro, Cristina Núñez, Rufina Bastida, José Luis Capelo, Carlos Lodeiro

**Affiliations:** [a] BIOSCOPE Group, REQUIMTE-CQFB, Chemistry Department, Faculty of Science and Technology, University NOVA of Lisbon2829-516, Monte da Caparica (Portugal) E-mail: cristina.nunez@fct.unl.ptcle@fct.unl.pt; [b] Ecology Research Group, Department of Geographical and Life Sciences, Canterbury Christ Church UniversityCT1 1QU, Canterbury (UK); [c] Inorganic Chemistry Department, Faculty of Chemistry, University of Santiago de Compostela15782 Santiago de Compostela (Spain)

**Keywords:** 1D nanochains, self-assembly, gold, mercury, nanoparticles, silver

## Abstract

Polyamine ligands are very versatile compounds due to their water solubility and flexibility. In the present work, we have exploited the binding ability of a polyamine molecular linker (**L**^2−^) bearing different functional groups, which favors the self-assembling of silver nanoparticles (AgNPs) and gold nanoparticles (AuNPs) into 1D nanochains in aqueous solution. The chainlike assemblies of AuNPs and AgNPs were structurally stable for a long period of time, during which their characteristic optical properties remained unchanged. The mechanism of AuNPs and AgNPs chain assembly associated with the induction of electric dipole–dipole interactions arising from the partial ligand exchange of surface-adsorbed citrate ions by (**L**^2−^) was investigated. UV/Vis spectrophotometry and transmission electron microscopy (TEM) were used to determine timedependent structural changes associated with formation of the 1D nanoparticle structures. Finally, the sensing of Hg^2+^ in aqueous solution using AgNPs@(**L**)^2−^ and AuNPs@(**L**)^2−^ assemblies was also carried out in aqueous solution.

## Introduction

Noble metal nanoparticles (MNPs) have been intensively pursued in recent years, not only for their fundamental scientific interest[1] but also for their technological applications, ranging from analytical sensors to catalysis and fuel cells.[2] Recently, the attention paid to 1D nanomaterials has been increasing significantly, because of the need to fabricate alternative functional 1D nanostructures for applications in the fields of nanoelectronics and nanobiotechnology,[3] due to the fact that they can act as interconnects between functional nanoscale components.[4]

Several experimental routes have been recently proposed to efficiently self-assemble preformed MNPs into 1D chains: methods involving hard,[5] polymeric[6] or surfactant-based[7] templates, molecular recognition,[8] specific functionalization,[9] and surface- or solvent-induced phase separation[10] have been successfully demonstrated.[11] Chains of MNPs also have been prepared by using linear macromolecular templates, such as, DNA,[12, 13] peptide,[14, 15] insulin fibrils,[16] protein fibrils[17, 18] or carbon nanotubes.[19]

The growth mechanism of self-assembled metal nanostructures using (macro)molecular ligands has been also reported to exhibit common features with molecular step-growth polymerization.[20] Similar to functional monomers, metal nanostructures assemble to form chains. The assembly was performed by small molecules (<2 nm), called molecular linkers, that contain at least two reactive ending groups, capable of attaching to a solid surface by chemisorption (thiol, amine) or interacting electrostatically with other functional groups (hydroxy, carboxyl, amine) present on the surface of nanoparticles (NPs).[20] The governing factor in linker-mediated assembly of MNPs is the equilibrium between the attractive and repulsive forces.[21] In particular, fabrication of anisotropic 1D noble MNP chains to obtain integrated optics operating below the diffraction limit of light has attracted much attention.[22]

Stellaci and co-workers[10a] have introduced anisotropic properties on ligand-stabilized AuNPs. Face-centered cubic (fcc) metallic NPs exhibit no intrinsic electric dipole, however, heterogeneities in surface charge and polarity, associated for example with the non-uniform spatial distribution of capping ligands on different crystal faces,[23] or nanophase separation in mixed-ligand stabilization layers,[24] are possible driving forces for anisotropic self-assembly.[25] In the case of spherical NPs, controlling the surface chemistry of the fabricated NPs allows the creation of an anisotropic ligand organization.[26] Enthalpy minimization, is obtained by promoting dipole alignment and reducing interdipole distances through the formation of linear chains of single NPs. This facilitates the orientation of specific interactions in one direction, which helps directing the selfassembly into 1D arrays. The self-assembly of the NPs into a well-defined 1D array is also influenced by interparticle chemical bonding, hydrogen bonding, van der Waals interactions, electrostatic forces, or any combination of these forces. In addition, entropy can be maximized at finite temperature by introducing some disorder in the linear chain, which corresponds to the incorporation of branching junctions and to chain reticulation that should be favored at elevated temperature.

Aggregation of NPs induces variations in absorption spectra accompanied by significant color changes of solutions.[27] Similar color changes can be observed upon the addition of an analyte, which initiates the aggregation of noble MNPs, and this feature can be used for permitting their industrial application in biosensing, immunological, and biochemical investigations.[28, 29] In the particular case of AgNPs, the geometrical shape also plays an important role in determining plasmon resonance properties.[30] For example, triangular, pentagonal, and spherical silver particles are colored red, green, and blue, respectively. Consequently, it is important to develop approaches that can manipulate NPs into different shapes and dimensions.

While many studies have tackled the synthesis and characterization of gold dimers and networks with peculiar plasmon resonance behavior,[31] organization of AgNPs in 2D superlattices is less common.[32, 33] For example, Chang et al. reported a variety of 1D- and 2D-nanostructured assemblies formed from AgNPs by variations in pressure, temperature, and time in supercritical water (SCW) without the need for any external linking agents.[34]

To follow our interest in new emissive materials, and functionalized NPs and to explore their applications,[35] herein, we investigate the mechanism of AuNP and AgNP chain assembly associated with the induction of electric dipole–dipole interactions. The nanoassembly capacity arises from the partial ligand exchange of surface-adsorbed negatively charged citrate ions, by covalently bound neutral molecular ligand **L** to produce a final mixed-ligand surface layer.

We show that exchange of surface adsorbed citrate with **L**, results in the formation of chain-like superstructures with topological features that are dependent on the extent of surface-ligand substitution. We determine the time-dependent structural changes associated with the formation of 1D NP superstructures. Morphological and optical characteristics of various nanostructures were investigated by TEM and UV/Vis.

## Results and Discussion

### Formation of chainlike structures from AgNPs and AuNPs

**Scheme 1:**
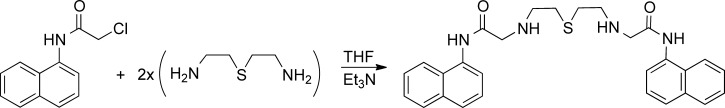
Synthetic route to compound **L**.

1D metallic silver or gold nanostructures, can be obtained by exploiting the binding ability of the linear polyamine molecular probe **L** in water (see chemical structure in Scheme 1). The donor atoms presented in the structure of compound **L** could be responsible for the formation of NP chainlike aggregates and the partial removal of the citrate ion from the starting metal nanoparticle (MNP) surface. This method is similar to that reported by Zhang et al.[36, 37] and it is different to that in which the assembly was induced by electrostatic interactions and the disassembly was labile in the presence of stronger chelating agents.[38]

In that case, firstly, we assumed that the level of ionization (protonation-deprotonation of functional groups) of potential modifying compounds, might play a role in the process of MNP functionalization, and consequently, could affect the stability of their dispersions. Therefore, the pH of the mixture was modified with a NaOH solution, to an approximate value of pH 12, which leads to the change in the linker ionization degree. After the addition of base to a solution of **L**, this compound was dissociated bearing negative charges **L**^2−^ because of deprotonation of the amine groups. Second, we assumed that the chemisorption of the linker on the AuNP surface could take place through the sulfur atom and/or by deprotonated amine groups, leading to the modification of the *ζ* potential from *ζ*_0_ (initial potential) to *ζ* (potential that determines the equilibrium between attractive and repulsive forces).

Finally, it is well known that changing the medium surrounding the nanoparticles (NPs) for another medium, having a markedly diﬀerent refractive index, strongly alters the surface plasmon resonance (SPR) band of the NPs in the UV/Vis spectrum. The position, intensity, and shape of SPR band strongly depends on the dielectric constant of surrounding medium, the size and shape of NPs as well as the electronic interaction between the stabilizing ligand and NP.[39] Therefore, UV/Vis absorption spectroscopy is an important analytical tool to probe the stability, surface chemistry, and aggregation behavior of AgNPs and AuNPs.

### Formation of chainlike structures from AgNPs

#### Spherical shape

**Figure 1:**
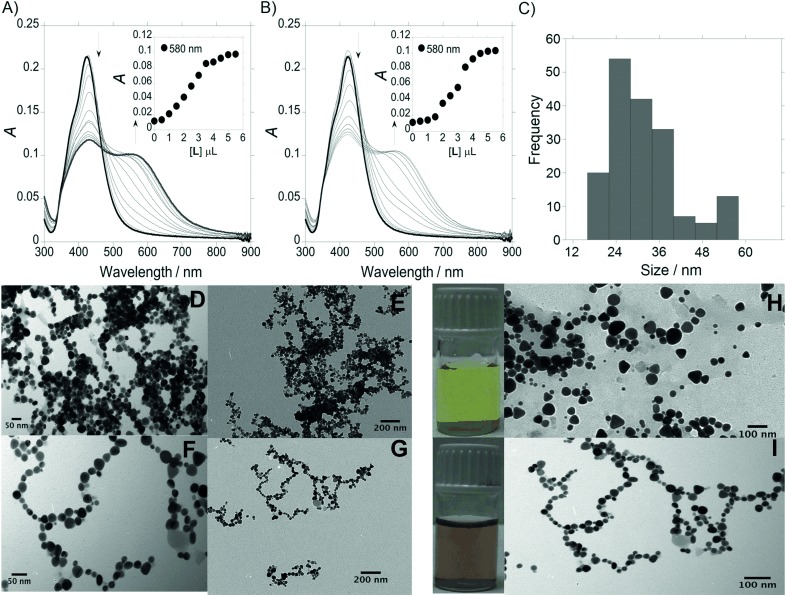
UV/Vis absorption spectra of spherical AgNPs@citrate during titration to A) AgNPs@**L** and B) AgNPs@(**L**)^2−^. C) Size distribution diagram for AgNPs@(**L**)^2−^. TEM images of silver nanowire formed from D,E) AgNPs@**L** and F,G) AgNPs@(**L**)^2−^. Visual color changes and TEM images obtained H) before and I) after the addition of **L**^2−^ to an aqueous solution of spherical AgNPs@citrate.

Initial characterization of spherical AgNPs prepared by citrate reduction of a silver nitrate solution revealed an absorption maximum (*λ*_max_) of the SPR peak for single particles at ≍420 nm (transverse SPR band; Figure 1 A,B). Analyses of TEM imaging shows that the prepared citrate-capped silver NPs (AgNPs@citrate) are nearly monodisperse spheres with an average size of about (25±3) nm. The AgNP concentration was estimated to be 7.8×10^−10^ in terms of molar concentration. This value was obtained taking into account that the entire mass of silver in AgNPs employed for colloidal dispersion preparation was fully transformed into NPs.

Figure 1 A and 1 B show the absorption titration of spherical AgNPs@citrate with compound **L** and **L**^2−^, respectively. As mentioned above, because AgNPs@citrate are highly dispersed in solution, the spectrum is characterized by a single SPR band at ≍420 nm (transverse SPR band). AgNPs@**L** and AgNPs@(**L**)^2−^ exhibit two absorption bands, at 420 nm and a new band at ≍580 nm, respectively. In both cases, the appearance of the second band (longitudinal SPR band) is a clear evidence of the assembly of AgNPs in solution, and a color change from yellow (Figure 1 H) to deep red (Figure 1 I) was also observed.

Chainlike structure formations of AgNPs in aqueous solution were induced in both cases by **L** and **L**^2−^, with the contact between AgNPs being easier at higher concentrations of **L** and **L**^2−^. The *ζ* potential distribution plays the major role in linker-mediated self-assembly of MNPs, and the development of linear chains and branched chain network is directed by the fact that the electrostatic double layer is rearranged around the dimers and becomes anisotropic.

The replacement of citrate ions most probably takes place after the addition of the negatively charged ligand **L**^2−^. In that case, the amount of negative charge on the surface of NPs does not decrease significantly, and as a result, a slight change of the *ζ* potential was observed from *ζ*_0_≍−33.5 mV cm^−1^ to *ζ*_0_≍−27.8 mV cm^−1^. The formations of chain-like superstructures with topological features are dependent on the extent of surface-ligand substitution. The replacement of citrate ions of the AgNPs surface by **L**^2−^ probably induces less electric dipole–dipole interactions, observing that the 1D NP assembly takes place less prominently (Figure 1 F,G) compared with that observed with **L** (Figure 1 D,E).

In that case, IR spectra were recorded to demonstrate the replacement of citrate ions of the AgNP surface by polyamine ligand **L**. The IR spectrum of compound **L** shows peaks characteristic of the carbonyl group at 1676 cm^−1^ and the 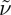
(C=C) stretching mode at 1436 cm^−1^ (see Figure S1, Supporting Information). In addition, a peak at 3262 cm^−1^ is observed due to the 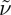
(N–H) stretching mode. A decrease in the intensity of the 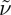
(C=O) band was observed in the IR spectra of AgNPs@**L** in comparison with **L**. The most interesting part of the spectrum is the region from 3000 to 3200 cm^−1^ due to 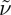
(N–H) stretching modes. The IR spectrum for AgNPs@**L** is very different from the spectrum of **L** in the same region. These results suggest the interaction of **L** with the MNP surface through the carbonyl and amine groups, confirming the replacement of the citrate ions from the NP surface.

#### Triangular shape

**Figure 2:**
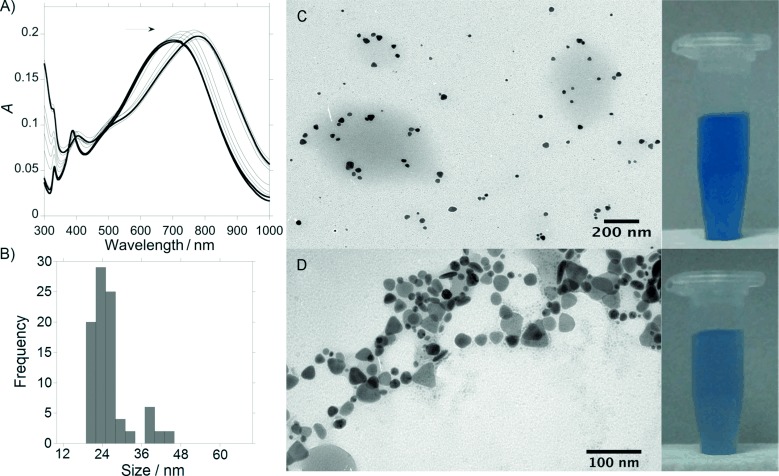
A) UV/Vis absorption spectra of triangular citrate@AgNPs during titration to AgNPs@(**L**)^2−^ and B) size distribution diagram for AgNPs@(**L**)^2−^. Visual color changes and TEM images obtained C) before and D) after the addition of **L**^2−^ to an aqueous solution of triangular AgNPs@citrate.

Because better results were obtained after titration of spherical AgNPs@citrate with the deprotonated ligand **L**^2−^, we used the same method with the triangular AgNPs@citrate. The formation of triangular AgNPs@citrate in aqueous solution with sizes in the range of (64±10) nm (see Figure S2, Supporting Information) was confirmed by the presence of the blue SPR band at ≍700 nm in the UV/Vis spectrum (Figure 2 A). As shown in Figure 2 C, the triangular AgNPs@citrate were well dispersed in milli-Q water before adding **L**^2−^, and they remained well separated on the TEM grid. After the addition of 5 μL of **L**^2−^ (1.10^−3^ m) to a solution of triangular AgNPs@citrate in aqueous solution, a redshift in the absorption band was observed to ≍780 nm, due to the formation of chains in which **L**^2−^ ions played the role of connectors between the AgNPs (Figure 2 D). As shown in Figure 2 C,D, a slight color change from intense to pale blue was also observed.

### Formation of chainlike structures from AuNPs

As a model experiment, we also used citrate-capped spherical gold NPs (AuNP@citrate) with a hydrodynamic diameter of (20±5) nm (see Figure S3, Supporting Information). The self-assembly was performed using a AuNP@citrate solution with a *C*_NP_=3.7×10^−9^ m (2.2×10^15^ particle L^−1^) and a concentration of 1.6×10^−6^ m for **L**^2−^ (≍pH 12) (*C*_L_/*C*_NP_≍4.5×10^2^).

**Figure 3:**
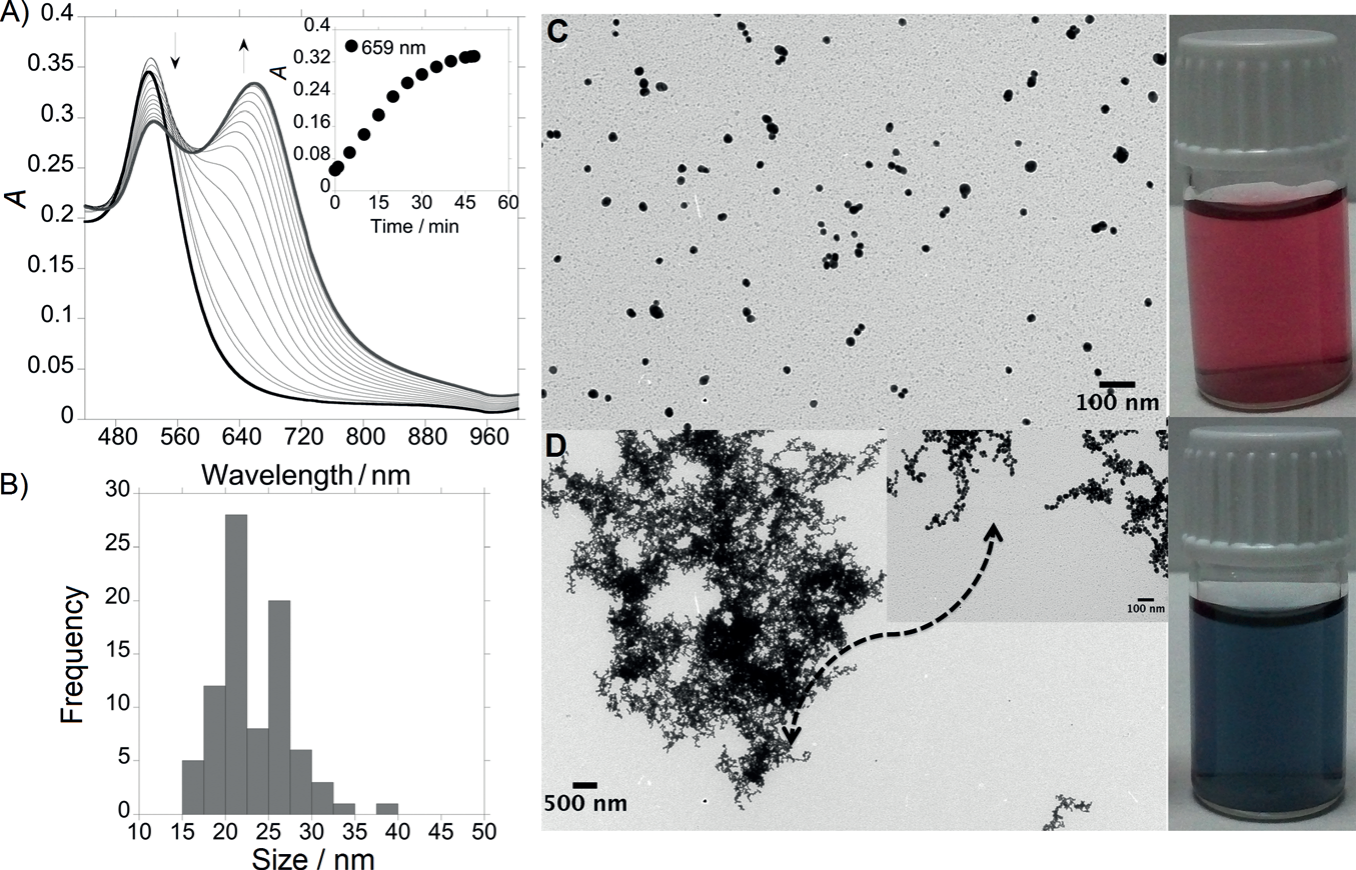
A) UV/Vis absorption spectra recorded in real-time (one spectrum per minute) during the self-assembly of AuNPs@citrate by **L**^2−^ to form AuNPs@(**L**)^2−^. B) Size distribution diagram for AuNPs@(**L**)^2−^. Visual color changes and TEM images of C) AgNPs@citrate and D) gold nanowire formed from AuNPs@(**L**)^2−^.

As we mentioned above, the assembly of AuNPs has a significant effect on the optical properties of the NPs, reflected by a dramatic change of the UV/Vis extinction value of the SPR band. As shown in Figure 3 A, single spherical AuNPs were characterized by an extinction transverse plasmon band at *λ*_1_≍520 nm.[40] After addition of the linker **L**^2−^, a second low-energy longitudinal surface plasmon band (*λ*_2_) appears at higher wavelengths (630–710 nm), which is a result of the plasmonic coupling of linearly assembled NPs. The position of this longitudinal surface plasmon band (*λ*_2_) could be modified in function of the topological distortions in the chains (Y-junction, zigzag defects, loop domains) in place of a strictly linear assembled superstructure. The second band shifts with time toward higher wavelengths and its intensity (*I*_*λ*2_) increases. As the interparticle spacing decreases, the first peak becomes weaker, while the second peak intensifies and shifts to longer wavelengths. The wide range of different chain morphologies observed in the extended networks accounts for the broadness of the emerging longitudinal plasmon band and the absence of isosbestic points in the time-dependent spectra.[15a]

The spectral change associated with the progressive aggregation of the NPs into chains and branched networks was also demonstrated by TEM (Figure 3 C,D). Consequently, the number of single NPs progressively decreases, leading to a decrease in the intensity of the first peak (*I*_*λ*1_).

The *ζ* potential of the original AuNPs@citrate solution was *ζ*_0_≍−33.5 mV cm^−1^, which is high enough (in absolute value) to keep the NPs electrostatically stable, and avoid aggregation due to repulsive forces between the negatively charged citrate ions. The use of citrate permits a controlled ligand exchange of the ions adsorbed on the surface. This spontaneous assembly is attributed to the electric dipole formed by the anisotropic organization of the ligands on the surface of the NP.[41] After the addition of **L**^2−^, a destabilization of the system was obtained with a value of potential *ζ*≍−27.0 mV cm^−1^. The effect of amine functionality on binding to the surface of AuNPs has been investigated,[42] and recently Chegel et al. reported experimental and theoretically studies about the cooperative functionalities of amine and thiol groups for aggregation of AuNPs.[43] However, some authors have claimed that one type of amine group can readily bind to Au colloids, whereas others cannot.[44] In that case, an efficient surface displacement of citrate ions on the presence of **L**^2−^ could take place due to a cooperative functionality of the deprotonated amine groups, and the sulfur atom capable of forming stable chemical bonds with gold atoms.

In the absence of linker molecules, the aggregation of gold nanospheres could be mainly caused by van der Waals attraction, yielding random and irregular geometries and usually leading to rapid precipitation. The presence of a functional linker as **L**^2−^ in the surface of NPs minimizes the effect of van der Waals attractions by significantly increasing long-range interactions represented by electrostatic forces.[45] Experimentally speaking, the *ζ* potential distribution seems to play the major role in linker-mediated self-assembly of AuNPs. As shown in Figure 3 C,D, a color change from intense red to blue was also observed.

In this particular case, the chainlike-structured AuNPs@(**L**)^2−^ were observed after no longer reaction time (45 min) due to connection between one particles with other NPs from solution. These superstructures remained unchanged even for prolonged incubation times such as two weeks.

### Interaction of AgNPs@(L)^2−^ and AuNPs@(L)^2−^ with Hg^2+^

**Figure 4:**
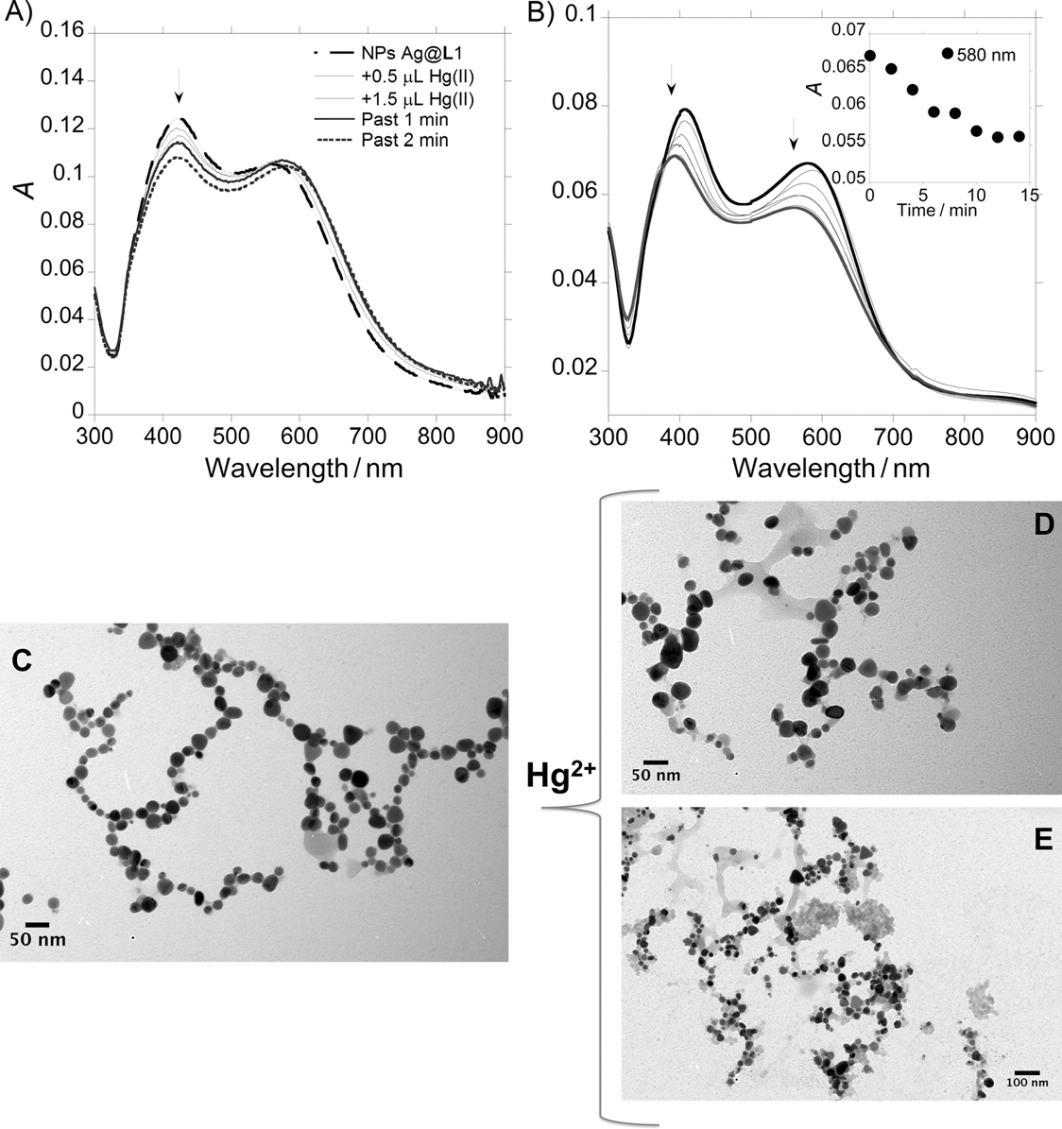
A) Spectrophotometric titration of spherical AgNPs@(**L**)^2−^ with the addition of increasing amounts of HgCl_2_ in aqueous solution. B) Modification with time in the absorption spectra of AgNPs@(**L**)^2−^ with the addition of 6 μL of HgCl_2_ (1:1 L/M). TEM images of an aqueous solution of spherical AgNPs@(**L**)^2−^ C) before and D,E) after the addition of HgCl_2_ (1 equiv, [HgCl_2_]=1.10×10^−3^ m).

**Figure 5:**
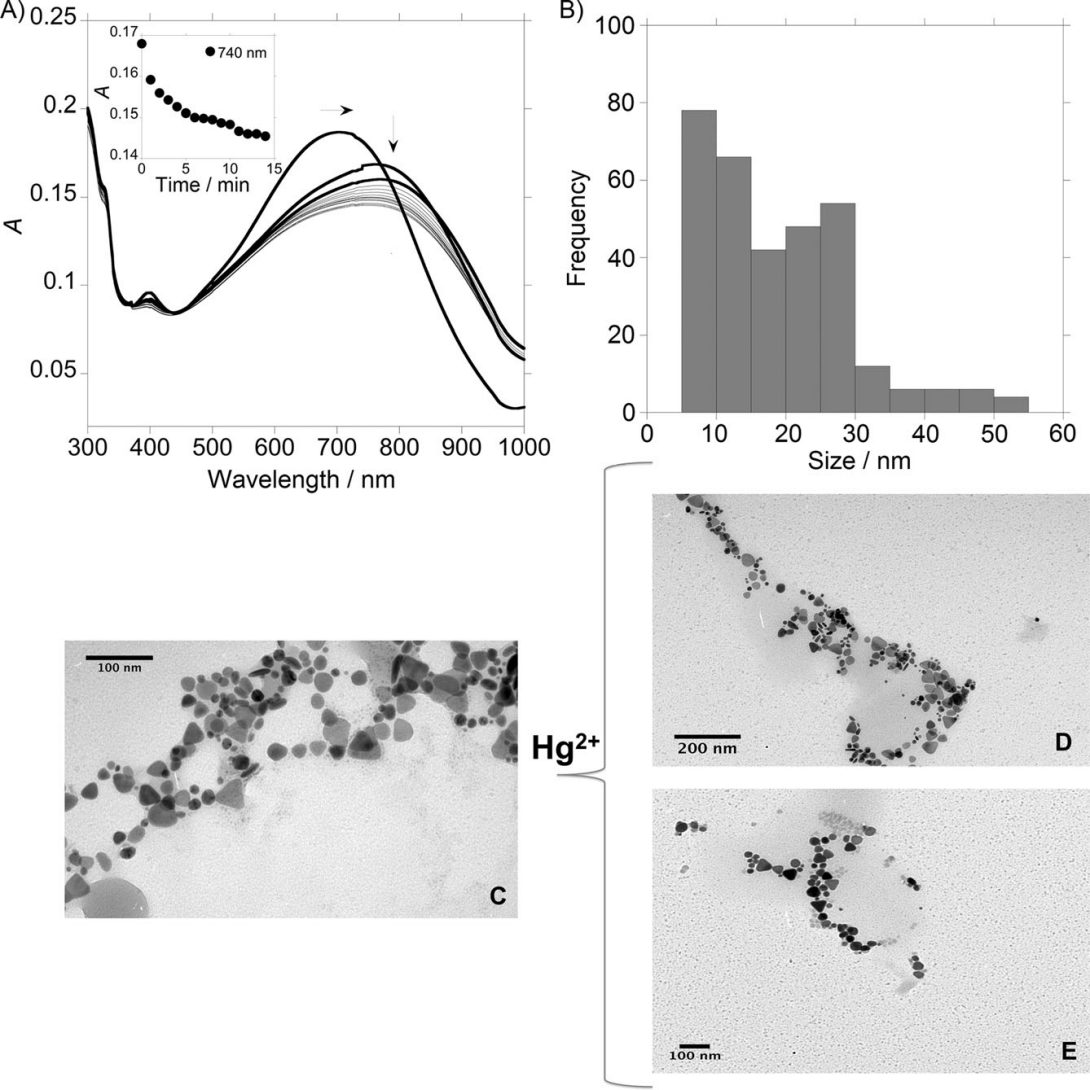
A) Modifications with time in the absorption spectra of triangular AgNPs@(**L**)^2−^ with the addition of HgCl_2_ (22 μL, 1:1 L/M). B) Size distribution diagram for an aqueous solution of triangular AgNPs@(**L**)^2−^ after the addition of HgCl_2_ (1 equiv, [HgCl_2_]=1.10×10^−3^ m). TEM images of an aqueous solution of triangular AgNPs@(**L**)^2−^ C) before and D, E) after the addition of HgCl_2_ (1 equiv, [HgCl_2_]=1.10×10^−3^ m).

Trying to apply the obtained nanostructures as optical effective nanochemosensors, the sensing of Hg^2+^ using the colloidal systems AgNPs@**L**^2−^ and AuNPs@**L**^2−^ was carried out in aqueous solution. Changes in the absorption spectra of the colloidal systems AgNPs@(**L**)^2−^ with spherical (Figure 4 A,B) and triangular (see Figure 5) shapes were observed after the addition of increasing amounts of Hg(NO_3_)_2_. In both cases, a blueshift of the SPR bands in the UV/Vis spectra suggest a continuous deformation of the chains, that is confirmed by TEM microscopy (Figure 4 D,E and Figure 5 D,E). The *ζ* potentials of the spherical and triangular AgNPs@(**L**)^2−^ solutions were *ζ*_spherical_≍−27.8 mV cm^−1^ and *ζ*_triangular_≍−19.1 mV cm^−1^, respectively. In both cases, after the addition of Hg^2+^, a destabilization of the systems was observed, and the value of *ζ* potentials changes to *ζ*_spherical_≍−22.5 mV cm^−1^ and *ζ*_triangular_≍−17.0 mV cm^−1^.

In view of these results, we can conclude that the destabilization of the spherical and triangular AgNPs@(**L**)^2−^ systems and the loss of the assembly, could be due to the more favorable interaction of **L**^2−^ with Hg^2+^ to that observed for **L**^2−^ with AgNPs. The exchange of **L**^2−^ adsorbed on the surface produces a decrease of the negative net charge in the NP surface, which causes an increase of the *ζ* potentials.

On the other hand, different results were obtained for linker **L**^2−^ adsorbed on the AuNPs surface because a more stable bond is formed compared with that obtained for **L**^2−^ and Hg^2+^, explaining that the AuNPs@(**L**)^2−^ assembly remains unchanged after the interaction with this metal ion.

The sensitivity of this nanoassembly system AgNPs@(**L**)^2−^ (spherical shape) toward Hg^2+^ was found to be comparable to the small fluorescent molecular system **L**. The value for the limit of detection (LOD) shows that the best candidate for the detection of this metal ion is system AgNPs@(**L**)^2−^, with the minimum amount of Hg^2+^ detectable being 8.3 ppm, whereas the LOD value with compound **L** was 19.0 ppm. The selectively of system **L** towards Hg^2+^ was also explored as shown in Figure S4 and S5.

## Conclusions

This study of 1D self-assembly of silver nanoparticles (AgNPs) and gold nanoparticles (AuNPs) demonstrates direct evidence of the cooperative interaction between the metal nanoparticles (MNPs) and the deprotonated polyamine compound **L**^2−^ at the nanoscale in aqueous solution.

The unexpected symmetry breaking that occurs when **L**^2−^ is added to an aqueous suspension of citrate-capped isotropic AgNPs and AuNPs was attributed to the ligand-mediated induction of a surface electrical dipole. At least three features of potentially modifying **L**^2−^ could influence citrate-stabilized AgNPs and AuNPs: (1) the presence of a sulfur atom, which can form covalent bonds with silver and gold atoms; (2) the presence of ionizable functional groups (amine); and (3) the charge (+/−) of ionizable functional groups. Electrostatic repulsion between AgNPs and AuNPs was progressively reduced, and the stability of the electric dipole associated with charge separation on the nanocrystal surface was potentially enhanced by spatial partitioning of **L**^2−^ and citrate-capping ligands. As a consequence, highly extended 1D NP assemblies in the form of discrete chains, bifurcated and looped chains, or interconnected chain networks are assembled spontaneously as the concentration of surface-adsorbed **L**^2−^ molecules increases.

Monoanionic compound **L**^2−^ causes shifts to the initial absorption spectra of AgNPs and AuNPs, and can be used for development of a surface plasmon resonance (SPR) chemical and biomolecular sensing platform because the interaction of citrate-stabilized AgNPs and AuNPs with the aforementioned compound is a very sensitive easy-to-visualize process.

The sensing of Hg^2+^ in aqueous solution using AgNPs@(**L**)^2−^ and AuNPs@(**L**)^2−^ was carried out. A destabilization of the system AgNPs@(**L**)^2−^ (spherical and triangular shape) and the loss of the assembly were observed due to the interaction of **L**^2−^ with Hg^2+^ is more favorable to that observed for **L**^2−^ with AgNPs. Linker **L**^2−^ binds to AuNP surfaces and forms a more stable bond compared with that obtained between **L**^2−^ and Hg^2+^, explaining that the AuNPs@(**L**)^2−^ assembly remains unchanged after the interaction with this metal ion.

## Experimental Section

### General

**Instrumentation**: Elemental analyses were carried out with Fisons Instruments EA1108 microanalyzer (Ipswich, UK) at the University of Vigo (CACTI), Spain. Infrared spectra were recorded in KBr windows using a JASCO FT/IR-410 spectrophotometer (Spain). ^1^H and ^13^C NMR were carried out on a Bruker Avance III400 at an operating frequency of 400 MHz for ^1^H NMR and 100.6 MHz for ^13^C NMR using the solvent peak as an internal reference at 25 °C. MALDI-MS analyses were performed with a MALDI-TOF/TOF MS model Ultraflex II (Bruker, Germany) equipped with nitrogen from the BIOSCOPE group. Each spectrum represents accumulations of 5×50 laser shots. The reflection mode was used, and the ion source and flight tube pressure were less than 1.80×10^−7^ and 5.60×10^−8^ Torr, respectively. The MALDI mass spectra of the soluble samples (1 or 2 μg μL^−1^) were recorded using the conventional sample preparation method for MALDI MS. One microliter was put on the sample holder on which the ligand had been previously spotted. The sample holder was inserted in the ion source. UV/Vis absorption spectra (220–900 nm) were performed using a JASCO-650 UV/Vis spectrophotometer (Oklahoma City, OK, USA) and fluorescence spectra on a HORIBA JOVIN-IBON Spectramax 4 (Irvine, CA, USA). All measurements were performed at 298 K.

**Limit of detection**: The limit of detection (LOD) for Hg^2+^ with the small fluorescent molecular systems **L** and the nanoassembly system AgNPs@(**L**)^2−^ (spherical) were performed having in mind their use for real ion detection and for analytical applications. The LOD was obtained using Equation 1:


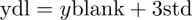
(1)

where ydl=signal detection limit and std=standard deviation.

**Concentration determination**: Assuming a spherical shape and uniform face centered cubic (fcc) structure, the molar concentrations of the silver nanoparticle (AgNP) and gold nanoparticle (AuNP) solutions was calculated using Equations 2 and 3.[46]


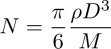
(2)

where *N* is the number of atoms per AgNP or AuNP, *ρ* [g cm^−3^] is the density of face centered cubic (fcc) silver (10.5 g cm^−3^) of gold (19.3 g cm^−3^), and *M* [g mol^−1^] is the atomic mass of sliver (107.86 g mol^−1^) or gold (196.97 g mol^−1^).


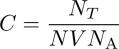
(3)

where *C* is the molar concentration of AgNPs or AuNPs, *N*_T_ is the total number of silver atoms added as AgNO_3_ or gold atoms added as HAuCl_4_, *N* is the number of NPs, *V* is the volume of the reaction solution in **L**, *N*_A_ is Avogadro’s constant (number of atoms per mole).

**Characterization of the assemblies of AgNPs and AuNPs**: We characterized the AgNPs, AuNPs and the chainlike assemblies of AgNPs and AuNPs using a number of optical tools including, transmission electron microscopy (TEM) and dynamic light scattering (DLS). To collect TEM images, the samples were prepared dropping 1 μL of the colloidal suspension onto a copper grid coated with a continuous carbon film and allowing the solvent to evaporate. TEM images were obtained using a JEOL JEM 1010F TEM operating at 200 kV. To perform the Fourier transformations, we used the Digital Micrograph (Gatan) software.[47] The NP size distributions were measured using the DLS system, Malvern Nano ZS instrument (Worcestershire, UK) with a 633 nm laser diode. We investigated the optical properties of these structures using a JASCO-650 UV/Vis spectrophotometer.

### Synthesis

**2,2′-((Thiobis(ethane-2,1-diyl))bis(azanediyl))bis(*N*-(naphthalen-1-yl)acetamide) (L)**: A solution of 20 % NaOH (3.4 g, 0.085 mol) was added to a stirred solution of 1-naphthylamine (9.34 g, 0.051 mol) in CH_2_Cl_2_ (30 mL). The mixture was cooled to 0 °C and chloroacetyl chloride (9.29 g, 0.083 mol) was added dropwise for 45 min. After stirring at 0 °C for 100 min, the mixture was allowed to warm to RT. The aqueous layer was separated and extracted with CH_2_Cl_2_ (2×25 mL). The combined organic phases were washed with HCl (5 % *v*/*v*), NaHCO_3_ (5 % *v*/*v*) and H_2_O, dried over MgSO_4_ and filtered to obtain a white solid. The crude product was purified by silica column chromatography and characterized as 2-chloro-*N*-(1-naphthyl)acetamide (74 %).

A solution of 2-chloro-*N*-(1-naphthyl)acetamide (439.34 mg, 2 mmol) in tetrahydrofuran (THF; 25 mL) was added dropwise to a solution of 2,2′-thiobis(ethylamine) (120 mg, 1 mmol) and triethylamine (202.24 mg, 2 mmol) dissolved in THF (50 mL) over 1 h in an ice bath. After the addition was completed, the reaction mixture was kept at reflux for 4 h. The solvent was removed in vacuo, and the residue was washed with H_2_O/CHCl_3_ (1:3 *v*/*v*; 4×20 mL). The resulting organic phase was dried in vacuo to give **L** as a pink powder (407.05 mg, 84 %): ^1^H NMR (500 MHz, CDCl_3_): *δ*=2.81 (t, 4 H), 3.12 (t, 4 H), 3.72 (m, 4 H), 5.84 (s, 2 H), 7.41–7.59 (m, 2 H), 7.72 (m, 2 H), 7.80 (m, 2 H), 7.92 (m, 4 H), 8.10 (m, 4 H), 10.35 ppm (s, 2 H); ^13^C NMR (500 MHz, CDCl_3_) *δ*=30.32, 54.85, 58.37, 120.69, 124.80, 125.73, 125.90, 127.20, 128.05, 128.19, 133.03, 133.56, 169.83 ppm; IR (KBr): 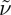
=1436 ((C=C)_ar_), 1676 (C=O), 3262 cm^−1^ (N–H); MALDI-TOF MS: *m/z* 487.21 [*M*+H]^+^; Anal. calcd for C_28_H_30_N_4_O_2_S: C 69.1, N 11.5, S 6.6, H 6.2, found: C 69.3, N 11.2, 6.4, H 6.6.

**Preparation of AgNPs**: Citrate-stabilized AgNPs of different shapes (spherical and triangular) were synthesized in aqueous solution following the Frank methodology.[48] For the synthesis of AgNPs with spherical shape, sodium citrate (2.0 mL, 1.25×10^−2^ m), AgNO_3_ (5.0 mL, 3.75×10^−4^ m), and H_2_O_2_ (5.0 mL, 5.0×10^−2^ m) were. After that, freshly prepared NaBH_4_ (2.5 mL, 5.0×10^−3^ m) was added. To obtain AgNPs with triangular shape, before the addition of NaBH_4_, KBr (40 μL, 1.0×10^−3^ m) was added to the solution. Once all reagents were combined, the resulting solutions were carefully swirled to fully mix the reactants. Almost immediately, the progression of the reaction becomes evident through the visual changes consistent with the growth of silver nanoprisms. Yellow and blue colors were observed for the spherical and triangular AgNPs, respectively (see Figure 1S, Supporting Information). Using Equations 2 and 3, *N*=30.70*D*^3^, and the resulting spherical AgNPs solution had a concentration of *C*_AgNP_=7.8×10^−10^ m with AgNPs of (25±3) nm size.

**Preparation of AuNPs:** Preparation of AuNPs was performed following the Turkevish method[49] through reduction of tetrachloroaurate ions (AuCl_4_^−^) by boiling in aq sodium citrate solution. HAuCl_4_⋅3 H_2_O (49.5 mg, 0.125 mmol) dissolved in nanopure H_2_O (125 mL; 18.2 MΩ cm) was added to a preheated solution of sodium citrate (12.5 mL, 1 wt %). The resulting solution was heated to 100 °C for 60 min and turned colorless before changing to violet and finally to ruby red. AuNPs obtained using this method appear as almost monodispersed globular structures with a size of (20±5) nm, which are stabilized by weakly bound citrate anions. Using Equations 2 and 3, *N*=30.896*D*^3^ for AuNPs and the resulting AuNPs solution was found to have a of *C*_AuNPs_=3.7×10^−9^ m.

**Chainlike assemblies of AgNPs and AuNPs**: We used the polyamine molecular probe **L**^2−^ to investigate the effect of the presence of different donor atoms in the AuNP assemblies and their optical properties. The formation of chainlike assemblies of AuNPs was controlled and modulated observing that the total formations were obtained by adding an acetonitrile solution of **L**^2−^ (5 μL, 1×10^−3^ m) into a suspension of AuNPs and AgNPs (circular and triangular) in nanopure H_2_O (≍10^−8^–10^−9^ m in 3 mL).

## Supplementary material

As a service to our authors and readers, this journal provides supporting information supplied by the authors. Such materials are peer reviewed and may be re-organized for online delivery, but are not copy-edited or typeset. Technical support issues arising from supporting information (other than missing files) should be addressed to the authors.
