# Supplementary material for: Polyamine Ligand-Mediated Self-Assembly of Gold and Silver Nanoparticles into Chainlike Structures in Aqueous Solution: Towards New Nanostructured Chemosensors
Source: ChemistryOpen. 2013 Aug 2;2(5-6):200–7. doi: 10.1002/open.201300023 (PMC3892197; doi:10.1002/open.201300023)
Supplement: Supplementary file 1 [file open0002-0200-SD1.pdf]

ChemistryOPEN

Supporting Information

© 2013 The Authors. Published by Wiley-VCH Verlag GmbH & Co. KGaA, Weinheim

Polyamine Ligand-Mediated Self-Assembly of Gold and Silver Nanoparticles into Chainlike Structures in Aqueous Solution: Towards New Nanostructured Chemosensors

Adrián Fernández-Lodeiro,[a] Javier Fernández-Lodeiro,[a] Cristina Núñez,*[a, b] Rufina Bastida,[c] José Luis Capelo,[a] and Carlos Lodeiro*[a]

open_201300023_sm_miscellaneous_information.pdf

Supporting Information

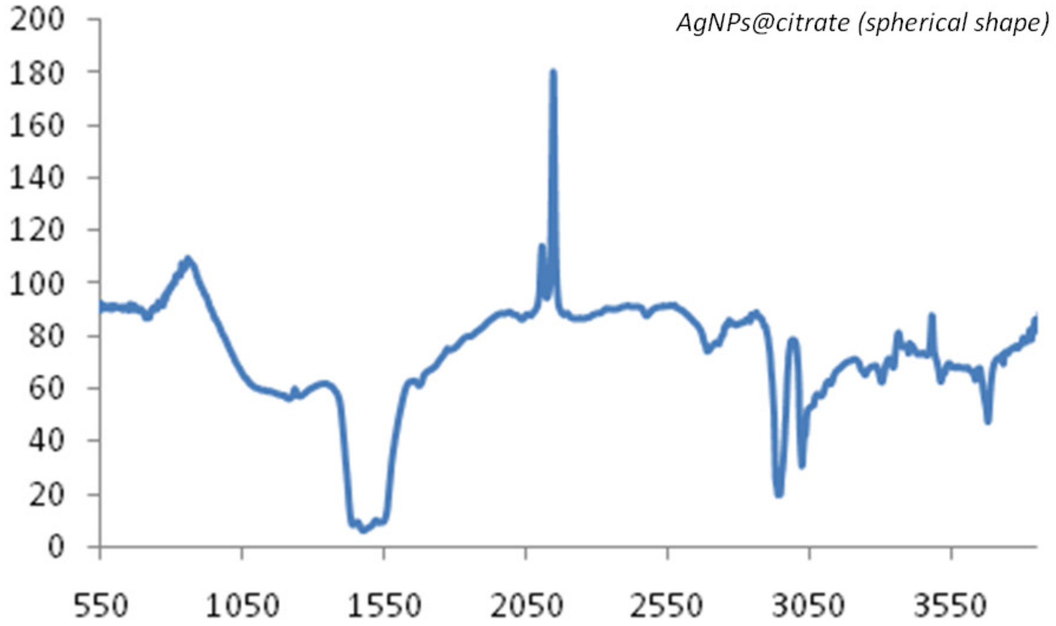

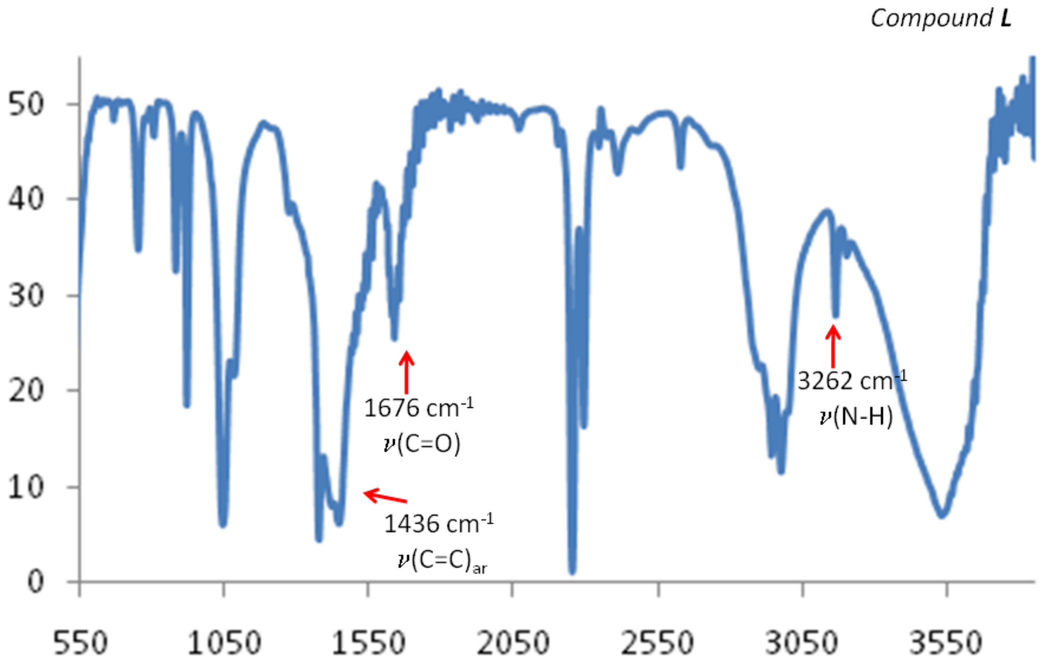

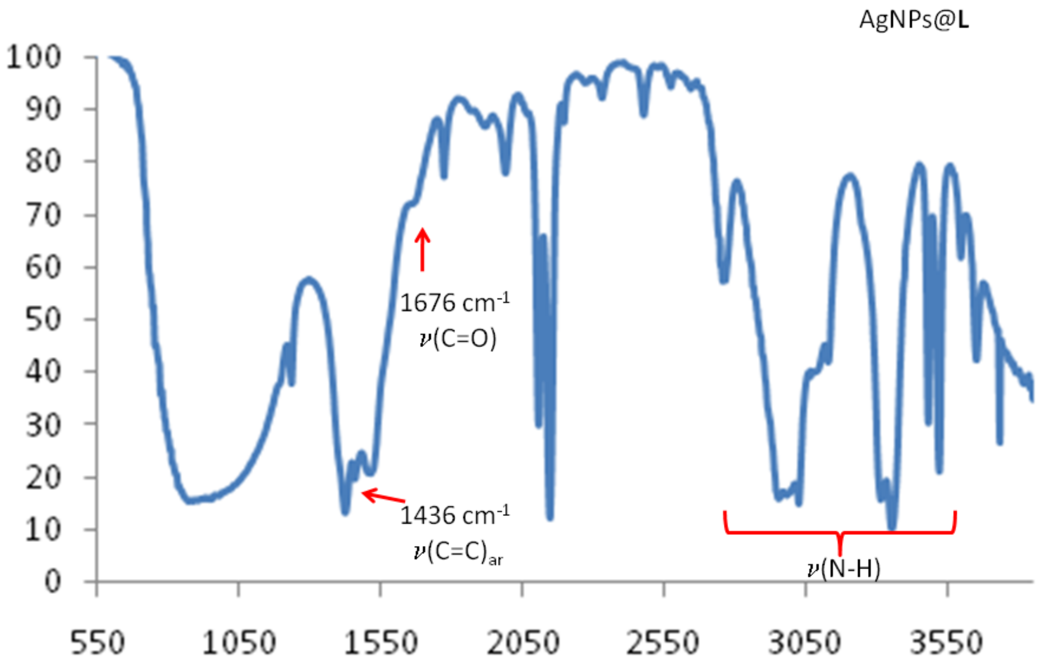

Figure S1. IR spectra of AgNPs@citrate, compound **L** and AgNPs@**L** in 550-4000 cm^{-1} region (x-axis = cm^{-1}, y-axis = intensity).

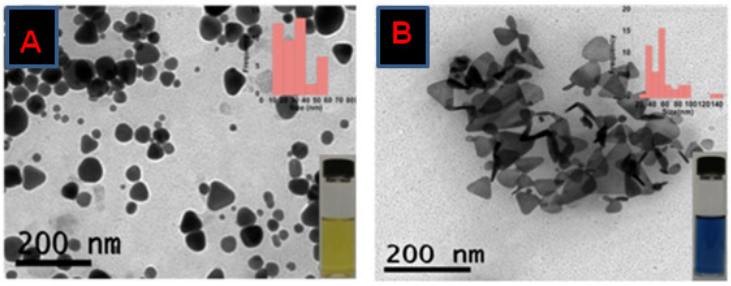

Figure S2. Transmission electron microscopy (TEM) images of AgNPs@citrate: (A) yellow (25 ± 3 nm; polydisperse quasispherical particles); (B) blue (64 ± 10 nm; average lateral dimension of triangular particles). The bar in each image corresponds to 200 nm.

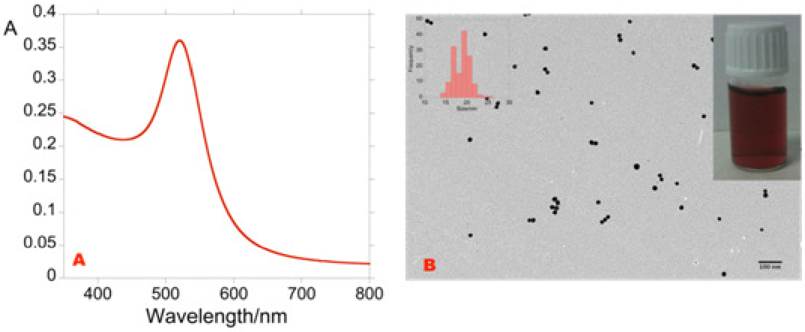

Figure S3. Absorption spectrum and transmission electron microscopy (TEM) images of AuNPs in aqueous solution.

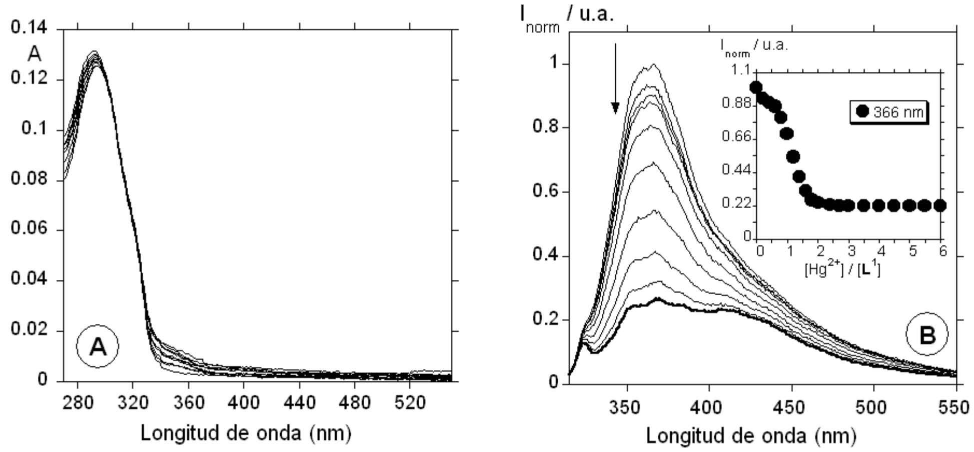

Figure S4. Spectrophotometric (A) and spectrofluorimetric (B) titrations of compound **L** as a function of added Hg^{2+} in absolute ethanol. The inset shows the normalized fluorescence intensity at 366 nm (B) λ_{exc} = 295 nm; λ_{em}= 366 nm, [**L**] = 1.00.10^{-5} M).

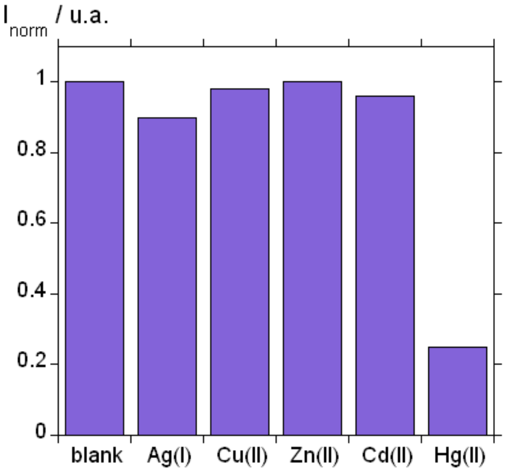

Figure S5. Normalized fluorescence intensity of receptor **L** in the absence and presence of one equivalent of different metal ions (Ag^{+}, Cu^{2+}, Zn^{2+}, Cd^{2+}, Hg^{2+}) in absolute ethanol. λ_{exc} = 295 nm; λ_{em}= 366 nm, [**L**] = 1.00.10^{-5} M).
